# Computed Tomography Angiography Diagnosis of a Congenital Intrahepatic Central Divisional Portocaval Shunt in an Arabian Filly

**DOI:** 10.1111/vru.70091

**Published:** 2025-09-25

**Authors:** Nick Cournoyer, Eric T. Hostnik, Rebecca Urion

**Affiliations:** ^1^ Department of Veterinary Clinical Sciences College of Veterinary Medicine The Ohio State University Columbus Ohio USA

**Keywords:** equine, hepatobiliary, microhepatica, portosystemic shunting, vascular

## Abstract

A 1‐month‐old Arabian filly presented for central neurologic signs that developed shortly after birth. Hyperammonemia and elevated bile acids were identified, prompting abdominal computed tomography angiography (CTA) evaluation on suspicion of congenital portosystemic shunting (PSS). CTA revealed an anomalous vessel connecting the intrahepatic central divisional portal branch and caudal vena cava, with microhepatica and bilateral renomegaly. Necropsy confirmed a single, congenital, and intrahepatic central divisional portocaval shunt. Imaging literature addressing PSS in foals is sparse, with the current case representing the first instance in which CTA alone was fully diagnostic of an intrahepatic shunt in a foal.

## 1 Signalment, History, and Clinical Findings

A 1‐month‐old Arabian filly (61 kg) was presented to The Ohio State University's Galbreath Equine Center for evaluation of neurologic signs. Lack of coordination was first noted 3 weeks prior to presentation, but otherwise, the filly appeared outwardly healthy. Upon initial assessment, hypermetria, proprioceptive ataxia, an inconsistent to absent menace response, and an unwillingness to elevate the head were observed. A Grade V/VI heart murmur was identified on auscultation, which was determined with an echocardiogram to be consistent with mild pulmonic stenosis and unlikely to explain the clinical presentation. No abnormal findings were appreciated on an initial screening abdominal ultrasound. Serum biochemistry values revealed hyperbilirubinemia (total bilirubin: 4.51 mg/dL, reference range: 0.5–2.5 mg/dL), hypoproteinemia (total protein: 3.50 g/dL, reference range: 5.3–7.2 g/dL), hypoalbuminemia (2.5 g/dL, reference range: 2.6–3.7 g/dL), and hypoglobulinemia (1.00 g/dL, reference range: 2.1–4.3 g/dL). Additional diagnostics revealed elevated bile acids (39 µmol/L, reference range: 4–12 µmol/L) and blood ammonia levels (115 µmol/L, reference range: 11–55 µmol/L). Abdominal computed tomography angiography (CTA) was pursued due to the high clinical suspicion of a portosystemic shunt (PSS).

## 2 Imaging, Diagnosis, and Outcome

Three‐phase abdominal CT angiography was performed under general anesthesia with the patient in sternal recumbency using a 64‐detector scanner (GE Revolution EVO, GE Healthcare). The CT parameters were as follows: 120 kVp, fluctuating mA with a maximum of 450 mA, 1.25 mm slice thickness, 1024 mm FOV, 512 × 512 matrix, and 0.984375 pitch. Reconstructions were made using default display parameters for window width (WW) and window level (WL) in a “STANDARD” soft tissue kernel (WW, 400 HU; WL 40 HU) and a “BONE” kernel (WW, 2500 HU; WL 250 HU). Positive contrast medium was administered through a jugular venous catheter (1.6 mL/kg with a total volume of 97 mL Omnipaque 300 Iohexol injection, Amersham Health Inc.). Transverse pre‐contrast as well as arterial, portal, and venous post‐contrast phases of the abdomen were acquired in helical fashion and reformatted into dorsal and sagittal planes. A 20‐s delay was utilized between the arterial and portal phases, and a 30‐s delay was utilized between the portal and venous phases.

**FIGURE 1:**
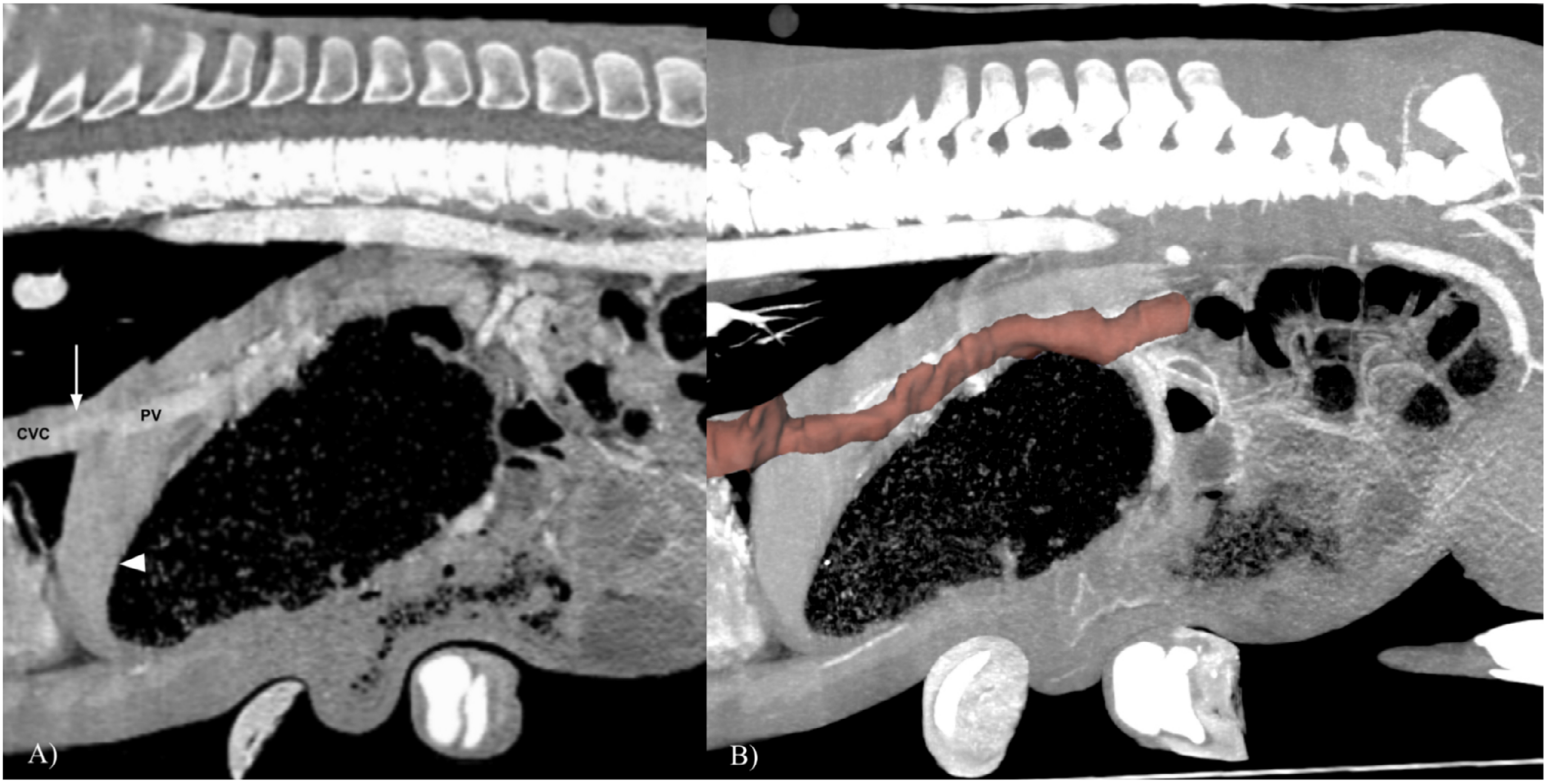
Computed tomography angiography portal phase sagittal reconstructions of the caudal thorax and cranial abdomen. (A) A contrast‐enhanced intrahepatic shunting vessel of the portal vein (PV) directly inserts into the caudal vena cava (CVC) at the level of the diaphragm (arrow). The liver is small (arrowhead). In (B), the abnormal shunting vessel is outlined in red. At the level of the right gastroduodenal vein, slice‐by‐slice manual segmentation of the portal vein overlaid on a maximum intensity projection (MIP) was performed to emphasize the outline of the vessel.

**FIGURE 2:**
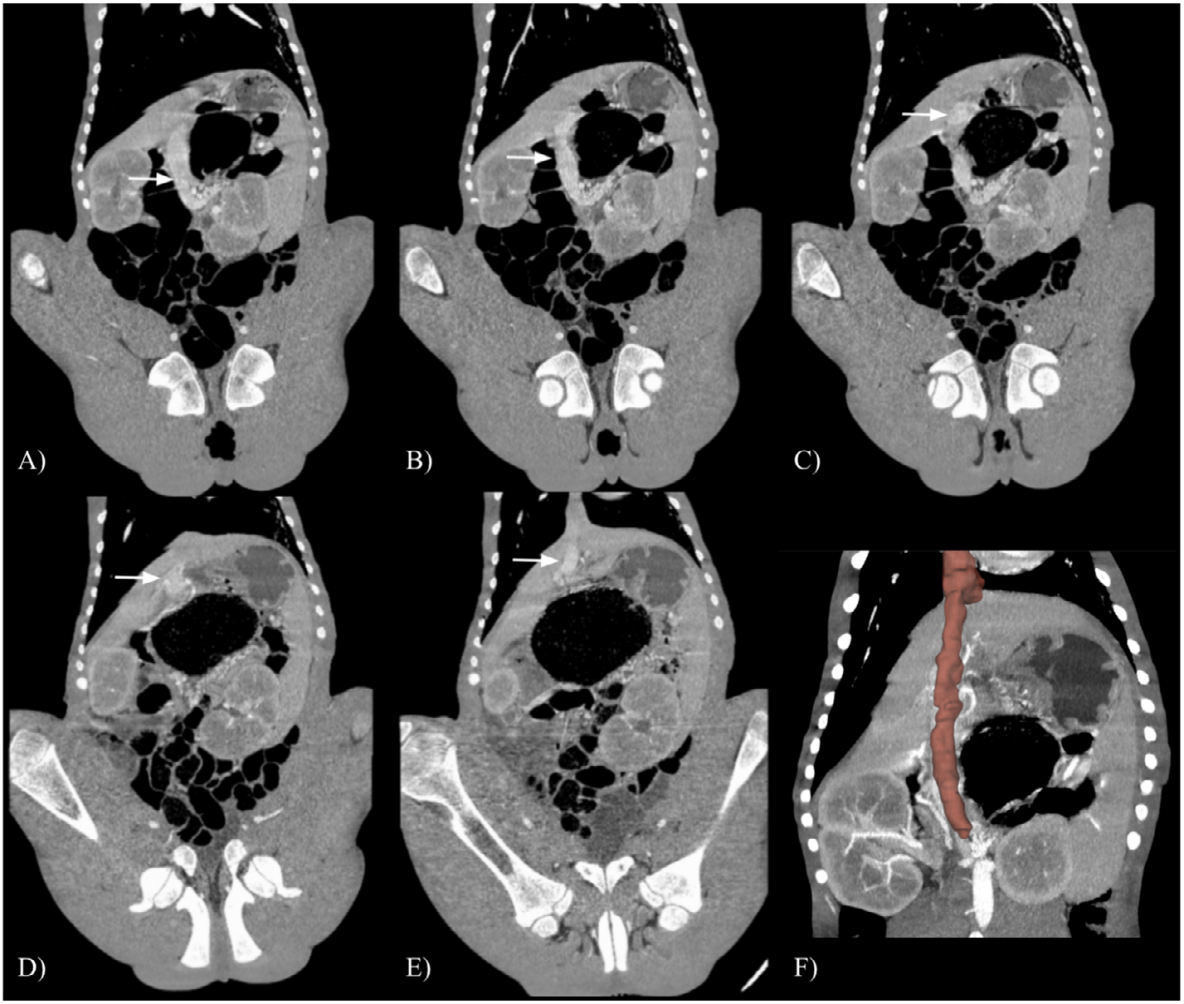
Computed tomography angiography portal phase dorsal reconstructions of the caudal thorax and abdomen. The letter labels (A–E) are on the patient's right, and the images are arranged from dorsal to ventral. The contrast‐enhanced central divisional branch of the portal vein (arrows) can be identified inserting directly into the intrahepatic caudal vena cava. The anomalous shunting vessel is outlined in red in (F). At the level of the right gastroduodenal vein, slice‐by‐slice manual segmentation of the portal vein overlaid on a maximum intensity projection (MIP) was performed to emphasize the outline of the vessel.

**FIGURE 3:**
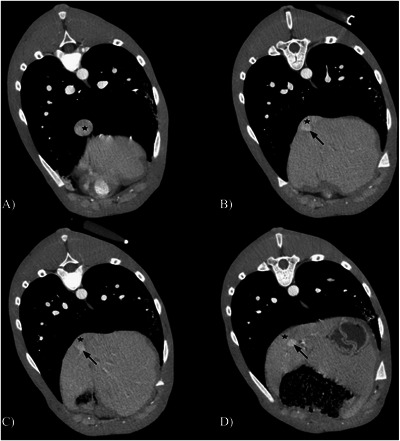
Transverse portal phase computed tomography angiography images of the intrahepatic central divisional portocaval shunt. The patient's right side is oriented to the left, and the individual panels (A–D) are arranged from cranial to caudal. An increase in the diameter of the caudal vena cava (asterisks) is present cranial to the insertion of the contrast‐enhanced shunting portal vessel (arrow).

**FIGURE 4:**
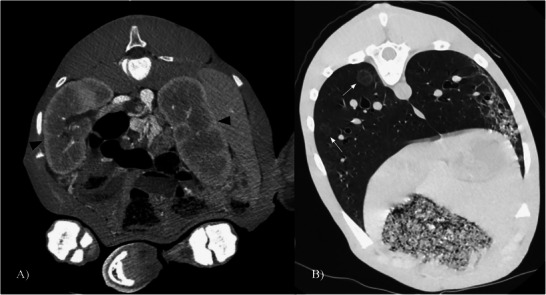
(A) Transverse portal phase computed tomography angiography image of the abdomen, demonstrating bilateral renomegaly secondary to the presence of portosystemic shunting (arrowheads). (B) Pre‐contrast, transverse lung window (WW: 1500 HU, WL: −600 HU) computed tomography image of the caudal thorax, demonstrating the presence of soft tissue nodules (arrows).

An anomalous vessel representing a single intrahepatic central divisional portocaval shunt was identified with CTA (Figure 1A,B). A normal portal vein (PV) was present, entering the liver at the porta hepatis, in combination with a normal right divisional portal branch extending into the right liver. However, the central divisional branch of the PV continued cranially through the liver and inserted directly into the ventral aspect of the intrahepatic caudal vena cava (CVC) at the level of the diaphragm (Figure 2A–F). The shunt vessel had a diameter of 7 mm at its confluence with the CVC. The prehepatic CVC was small and had decreased contrast filling, with an approximately 30% increase in diameter cranial to the insertion of the shunting vessel (Figure 3A–D). The liver was severely reduced in size, with the left side being worse than the right, and subjective bilateral renomegaly was present, collectively compatible with PSS (Figure 4A). There were also faint, round, and smoothly marginated multifocal soft tissue nodules present throughout the caudal lungs, measuring up to 1.5 cm in diameter (Figure 4B). Transcutaneous abdominal ultrasound was attempted but was unsuccessful due to the deep and central location of the shunting vessel, prohibiting vessel identification and Doppler interrogation of portal flow direction and velocity. After consideration of surgical treatment options, the owners elected to pursue humane euthanasia due to poor prognosis and the presence of concurrent congenital pulmonary stenosis. A postmortem examination confirmed the diagnosis of a single, congenital central divisional intrahepatic portocaval shunt. The pulmonary nodules noted were determined to be representative of pyogranulomatous pneumonia from an unconfirmed etiologic agent upon necropsy.

## 3 Discussion

A PSS is a vascular anomaly in which the portal venous circulation directly enters systemic circulation without communicating with the liver [1]. Although PSS identification has been firmly established in small animal medicine, there is far less documentation of the abnormality in the equine field. PSSs are classified on the basis of their connected vascular systems, relation to the liver, number of shunt vessels, shunt fraction, and presumed etiology (e.g., congenital or acquired) [2]. Extrahepatic PSSs are more commonly reported in foals, with only two previously documented cases of intrahepatic PSS, to the authors’ knowledge [3, 4]. Clinical signs in foals can be nonspecific but most consistently include central neurologic signs, likely due to secondary hepatic encephalopathy.

Partly due to the rare occurrence of PSSs in horses, PSS is an uncommon differential diagnosis in the absence of strongly suggestive lab work results [5]. Among the reported cases of confirmed PSS in foals, all patients shared common lab work findings of hyperammonemia and elevated bile acids, whereas the presence of hyperbilirubinemia was variable [6, 7]. Definitive diagnosis of PSS requires diagnostic imaging, and numerous imaging modalities and techniques have been used across species, including cranial mesenteric and intraoperative mesenteric portography, percutaneous splenoportography, ultrasonography, transcolonic and trans‐splenic portal Tc 99m scintigraphy, and the increasingly utilized CTA [1, 5].

Among documented equine PSS cases, imaging modalities utilized in the diagnostic workup in foals have predominantly consisted of transcutaneous ultrasonography, contrast mesenteric portography, and CTA [6]. Ultrasonography is the least invasive and costly, with added benefits including the capability of measuring portal flow velocity. However, transcutaneous ultrasonography in horses can be limited due to overlying gastrointestinal gas, limited hepatic windows, and acoustic shadowing from the lungs [8]. An added level of difficulty in identifying an intrahepatic PSS in comparison to an extrahepatic PSS in foals through abdominal ultrasound has also been suggested [9]. Although contrast mesenteric portography allows for visualization of the portal venous system on radiographs, the modality requires an invasive approach, including general anesthesia and celiotomy to gain appropriate venous catheter access for contrast medium injection [3].

In contrast, evaluation of PSSs by means of CTA is gaining traction due to the ability to yield a precise overview of portal vasculature in a timely and noninvasive manner [1, 10]. Multiphase scanning in combination with positive contrast medium administration allows for better recognition of vessels, and three‐dimensional vasculature models can be constructed to further evaluate morphologies and aid in surgical planning. CTA has also been reported to have a high sensitivity and specificity of 96% and 89%, respectively, in canine PSS detection [11]. As long as a patient's size is compatible with the CT gantry dimensions, reliable diagnostic insight can be expected, even when anomalous vessels are small or located deep within the abdomen and/or hepatic parenchyma. The current case demonstrates the effectiveness of the imaging modality as a standalone or adjunctive diagnostic tool in the identification of various PSS configurations. In only three other documented cases of the condition involving CTA in young horses, the diagnostic modality was definitive in identifying a congenital extrahepatic splenocaval shunt in a 1‐month‐old Dutch Warmblood colt, a left gastrocaval extrahepatic PSS in a 2‐week‐old pony foal, and an acquired transhepatic PSS in a 1‐year‐old Miniature Horse filly with suspected pyrrolizidine alkaloid toxicosis [7, 12, 13].

A congenital intrahepatic PSS, such as in the present case, may be less common than extrahepatic PSS in foals due to differences in embryonic development compared to other species. A significant portion of intrahepatic PSS cases are due to incomplete closure of the ductus venosus; however, compared to the current case, PSSs secondary to ductus venosus anomalies generally involve the left hepatic division [14]. For most species, the ductus venosus does not close until after birth, as seen with dogs, where closure occurs within 2–9 days of life [15]. Unlike other neonates, the ductus venosus closes completely in horses during gestation, eliminating a common risk factor associated with intrahepatic shunts [16]. Hereditary breed associations for congenital PSSs have been documented in dogs and cats [17], but no such associations have yet been documented in horses, likely due to low case numbers in this species. As this is now the second report of PSS in an Arabian or Arabian cross though [4], further work in this area is likely indicated.

Additional CTA findings of microhepatica and bilateral renomegaly in the present case were both considered secondary to, and are commonly reported features of, PSSs (Figures 1A and 4A). When portal blood bypasses the liver from a shunt, there is also a loss of trophic factor delivery from the gastrointestinal tract [18]. Without trophic factors vital for liver health and regeneration, hepatic hypoplasia ensues. The general pathophysiology of renomegaly with PSS is not as well‐defined, but proposed theories include compensatory metabolic mechanisms due to the lack of a functional liver or mechanisms of maintaining homeostasis and hepatic blood flow by increasing glomerular filtration rates and blood flow to the kidneys [19]. There is also a suspected association between the severity of portal blood shunting and the development of secondary renomegaly. Other findings that may be present in cases of PSS include urolithiasis, cachexia, enlarged tortuous hepatic arteries, periportal edema, and a reduction of portal enhancement downstream of the intrahepatic shunt origin [2].

Prior to postmortem examination, we initially hypothesized that the identified pulmonary nodules were representative of potential abscesses of hematogenous origin secondary to PSS (Figure 4B). There is no documentation of such pathology in horses to date, but PSS has been identified as a potential cause of septic physitis in canines due to bacteremia from the unfiltered blood bypassing the liver [20]. With the current case representing the first foal with PSS and coexistent pulmonary nodules despite the absence of respiratory signs, further observation and documentation of pulmonary lesions in future cases are warranted.

The current case is the first documented instance of an intrahepatic central divisional PSS in a foal diagnosed via CTA alone and further demonstrates the utility of CTA in providing a definitive in vivo diagnosis of PSSs in this patient population. With the case‐dependent variability of shunt morphology bringing uncertainty to the prognosis of each affected individual, the additional vascular visualization benefit of CTA allows for optimal prognostication and surgical planning and may lead to a decreased risk of complications in future procedures.

## Author Contributions

**Nick Cournoyer**: conception and design, acquisition of data, analysis and interpretation of data, drafting the article, revising article for intellectual content, final approval for the completed article, agreement to be accountable for all aspects of the work ensuring that questions related to the accuracy or integrity of any part of the work are appropriately investigated and resolved. **Eric T. Hostnik**: analysis and interpretation of data, revising article for intellectual content, final approval for the completed article, agreement to be accountable for all aspects of the work ensuring that questions related to the accuracy or integrity of any part of the work are appropriately investigated and resolved. **Rebecca Urion**: conception and design, acquisition of data, analysis and interpretation of data, drafting the article, revising article for intellectual content, final approval for the completed article, agreement to be accountable for all aspects of the work ensuring that questions related to the accuracy or integrity of any part of the work are appropriately investigated and resolved.

## Disclosure

Dr. Eric T. Hostnik is the Editor‐in‐Chief of Veterinary Radiology & Ultrasound. Dr. Hostnik recused himself from any decision about manuscript acceptance to this journal. Authors were anonymized for the review process. The manuscript/abstract was not presented before in any meeting or conference and has not been submitted to any other journal. A reporting checklist was not used on this occasion.

## Conflicts of Interest

The authors declare no conflicts of interest.

## Data Availability

The data supporting the results of this study are available from the corresponding author upon reasonable request.
